# Lack of epidermal growth factor receptor (*EGFR*)-activating mutations in triple-negative breast cancer in China

**DOI:** 10.1186/s13058-015-0628-6

**Published:** 2015-08-20

**Authors:** Wen-Ming Cao, Yun Gao, Xiao-Jia Wang

**Affiliations:** Department of Medical Oncology, Zhejiang Cancer Hospital, 38 Guangji Road, Hangzhou, 310022 China; Zhejiang Key Laboratory of the Diagnosis & Treatment Technology on Thoracic Oncology, Zhejiang Cancer Hospital, 38 Guangji Road, Hangzhou, 310022 China; Institute of Cancer Research, Zhejiang Cancer Hospital, 38 Guangji Road, Hangzhou, 310022 China

We read with interest the study by Teng and colleagues reporting a high frequency (11.4 %) of epidermal growth factor receptor (*EGFR*)-activating mutations in triple-negative breast cancer (TNBC) in a Singapore cohort [1]. In a separate study, *EGFR*-activating mutations were detected in 7.7 % (1 out of 13) of Chinese basal-like breast cancers [2]. Interestingly, this frequency ranged from 0 to 3 % of other Asian and Caucasian patients [3, 4]. These results indicate that the frequency of these mutations may vary according to geographic and ethnic differences, as reported in non-small cell lung cancer, and these mutations appear to be limited mostly to Chinese patients with TNBC. In this study, in-depth characterization of these mutations in Chinese TNBC was attempted on retrospective archival tissues.

Fifty freshly frozen specimens of TNBC from patients without neoadjuvant chemotherapy were randomly selected from Zhejiang Cancer Hospital, China, from 2010 to 2011. The specimens were confirmed to be estrogen receptor- and progesterone receptor-negative, and less than 1 % of tumor cells showed positive nuclear staining by an immunohistochemistry (IHC) assay. HER-2 negativity was defined as a score of 0 or 1+ by IHC assay or a *HER-2*/chromosome 17 ratio of less than 2 and fewer than 4 *HER-2* copies per nucleus by a fluorescence in situ hybridization assay. Written consent was obtained from all participants. This study was approved by the Research and Ethics Committee of Zhejiang Cancer Hospital.

Twenty-five *EGFR*-activating mutations (G719S, G719A, G719C, S768I, L858R, and L861Q and 19 mutations of exon 19-Del) were analyzed by an Amplification Refractory Mutation System (ARMS) assay by using an ADx EGFR29 Mutation Kit (Amoy Diagnostics, Xiamen, China). The ARMS assay is able to detect mutations with allele frequencies as low as 1 % [5]. Exons 18, 19, 20, and 21 were amplified by polymerase chain reaction assay, and all fragments were bidirectionally sequenced to screen for other mutations.

None of the *EGFR*-activating mutations was found by these two assays, but four single-nucleotide polymorphisms (SNPs) were identified by the sequencing assay (Table 1).

**Table 1:** Polymorphisms in exons 18, 19, 20, and 21 of *EGFR* in 50 triple-negative breast cancers

Exon	Position	Amino acid change	Minor allele frequency (number)	dbSNP	NNSplice		Human Splicing Finder
					Score ratio of donor site (SNP versus normal)	Score ratio of acceptor site (SNP versus normal)	
Intron 19	c.2283 + 103C > T	-	0.1 (10)	rs17290371	0.48:0.53	-	Creation of an intronic ESE site
20	c.2361G > A	Q787Q	0.18 (9)	rs1050171	0:0.43	-	Probably no impact on splicing
20	c.2457G > A	V819V	0.04 (1)	rs56183713	0.53:0	0.89:0.65	1. Creation of an exonic ESS site
							2. Alteration of an exonic ESE site
Intron 20	c.2470-68C > A	-	0.02 (1)	rs530416576	-	-	Creation of an intronic ESE site

*EGFR* epidermal growth factor receptor, *SNP* single-nucleotide polymorphism, *ESE* exonic splicing enhancer, *ESS* exonic splicing silencer

In silico prediction was performed to investigate the effect of the SNPs on gene splicing by using two programs: NNSplice 0.9 version (http://www.fruitfly.org/seq_tools/splice.html) and Human Splicing Finder (http://www.umd.be/HSF3/). All four SNPs, particularly the c.2457G > A SNP, were predicted to alter splicing by one or both programs.

In summary, none of the well-known *EGFR*-activating mutations was identified in our cohort. This suggests that TNBCs form a group of cancers with marked heterogeneity. Targetable mutations may be present and clinically helpful in only a limited number of Chinese patients with TNBC.

## Abbreviations

ARMS: Amplification Refractory Mutation System
EGFR: Epidermal growth factor receptor
IHC: Immunohistochemistry
SNP: Single-nucleotide polymorphism
TNBC: Triple-negative breast cancer
